# Endocannabinoids Block Headache and Anxiety Comorbidity via Two-Pronged Anterior Insular Projections

**DOI:** 10.34133/research.1031

**Published:** 2025-12-09

**Authors:** Jiang Bian, Yue-Hui Zhang, Li Yin, Jian-Feng Li, Xia Zhang, Xu-Feng Xu

**Affiliations:** ^1^ Institute of Brain Function and Diseases, Departments of Neurology and Psychiatry, West China Hospital of Sichuan University, Chengdu 610041, China.; ^2^ Neuropsychiatry Research Institute, School of Basic Medicine, The Affiliated Hospital of Qingdao University, Qingdao University, Qingdao 266000, China.; ^3^ Departments of Anesthesiology, Neurology and Clinical Research Center, Panzhihua Central Hospital, Panzhihua 617000, China.; ^4^ School of Chinese Materia Medica and Yunnan, Key Laboratory of Southern Medicinal Utilization, Yunnan University of Chinese Medicine, Kunming, Yunnan 650500, China.

## Abstract

The mechanism of and effective treatment for the headache, with the global prevalence of 52%, and its common anxiety comorbidity remain elusive. Here, we found that chronic isosorbide dinitrate (ISDN) injections induce c-fos expression in the anterior insula (AI), prelimbic cortex (PrL), and oval nucleus of the bed nucleus of the stria terminalis (ovBNST), suggesting their contributions to headache and anxiety comorbidity. This hypothesis is substantiated by our findings that chronic ISDN injection-induced headache and anxiety are blocked by inhibition of ventral AI (vAI)-PrL and dorsal AI (dAI)-ovBNST circuits, respectively. Headache and anxiety stimuli in chronic ISDN-injected mice markedly increase endocannabinoid (eCB) release at both glutamatergic vAI-PrL synapses and dAI-ovBNST synapses, indicating the role of eCB signaling in modulating headache and anxiety. Indeed, presynaptic knockdown of eCB hydrolase or presynaptic activation of cannabinoid type 1 receptors (CB1Rs) in vAI-PrL and dAI-ovBNST circuits separately alleviates headache and anxiety. A systemic application of eCB degradation enzyme inhibitors blocks chronic ISDN-induced headache and anxiety comorbidity, which are separately blocked by CB1R antagonist application in PrLs and ovBNSTs. Our findings reveal divergent counteracting effects of elevated eCB signaling in vAI-PrL and dAI-ovBNST circuits on comorbid headache and anxiety.

## Introduction

Anxiety is particularly prominent in psychiatric comorbidities of the headache, with the global prevalence of 52% [1,2]. This bidirectional relationship creates a self-perpetuating pathological loop that remarkably complicates therapeutic interventions [3–5]. Largely because the exact mechanisms underlying headache and anxiety comorbidity remain unclear, pharmacological treatments currently used in clinical practice often show limited and varied lasting effects on headache and anxiety comorbidity [6]. Therefore, uncovering the underlying mechanisms and identifying a single therapeutic target are crucial for preventing and treating this comorbidity.

Endocannabinoids (eCBs), predominantly 2-arachidonoylglycerol (2-AG) and anandamide (AEA), function through activity-dependent synthesis in postsynaptic neurons, release into synaptic clefts, retrograde activation of presynaptic cannabinoid type 1 receptors (CB1Rs), suppression of presynaptic release of neurotransmitters, and degradation by eCB hydrolase [7]. Emerging preclinical and clinical evidence positions the therapeutic potential of increased brain eCBs in multiple pathological pain and affective disorders [8,9]. Pharmacological agents, including CB1R agonists, monoacylglycerol lipase (MAGL) inhibitors (for 2-AG), and fatty acid amide hydrolase (FAAH) inhibitors (for AEA), have demonstrated efficacy in alleviating headache and anxiety-related behaviors [10–12]. Conversely, disrupting the eCB signaling by pharmacological blockade of CB1R or inhibition of eCB biosynthesis exacerbates headache and anxiety-like phenotypes [13–15]. Clinically, patients with eCB deficiency syndrome exhibit heightened headache susceptibility, suggesting involvement of eCBs in headache pathophysiology [16]. However, whether and how eCBs can treat, as a single therapeutic medicine, headache and anxiety comorbidity remains elusive.

Clinical evidence has revealed abnormalities in multiple brain regions in patients with chronic headache, including the insular [17–19] and prefrontal cortices [20], thalamus [21], hypothalamus [22], and limbic system [23]. However, the exact brain regions linking chronic headache and its comorbid anxiety remain poorly understood. The insular cortex, or insula, is a critical integration hub with abundant CB1Rs and high-density connectivity to an extensive network of multiple brain regions and is well established in regulating the sensory, affective, motivational, and cognitive processes [24]. The insula has 2 anatomical subdivisions, i.e., the anterior insula (AI) and posterior insula (PI), each with distinct neural connectivity and functions [25]. Pharmacological modulation of AI neurons produces analgesic and anxiolytic effects [26–28]. Intriguingly, AI neurons send output to multiple brain regions involved in pain or affective regulation, such as the oval nucleus of the bed nucleus of the stria terminalis (ovBNST) and prelimbic cortex (PrL) [29–31], suggesting the possible modulatory roles of AI-PrL and AI-ovBNST circuits in headache and anxiety comorbidity. Nonetheless, it is entirely unknown whether and how eCB signaling contributes to headache and anxiety comorbidity through AI-PrL and AI-ovBNST circuits.

In this study, we integrated behavioral assays, chemogenetic manipulation, tract-tracing methods, RNAscope in situ hybridization, and 4 projection- and synapse-specific eCB-targeted molecular probes to elucidate the contributions of eCB signaling in AI-PrL and AI-ovBNST circuits to the pathophysiology of comorbid headache and anxiety. Using a mouse model of chronic headache induced by repetitive intraperitoneal injections of isosorbide dinitrate (ISDN), we demonstrated the association of therapeutic effects of systemic eCB application on headache and anxiety comorbidity with increased eCB signaling and subsequent activation of AI-PrL and AI-ovBNST circuits and counteraction.

## Results

### Headache and anxiety model establishment

**Fig. 1.**
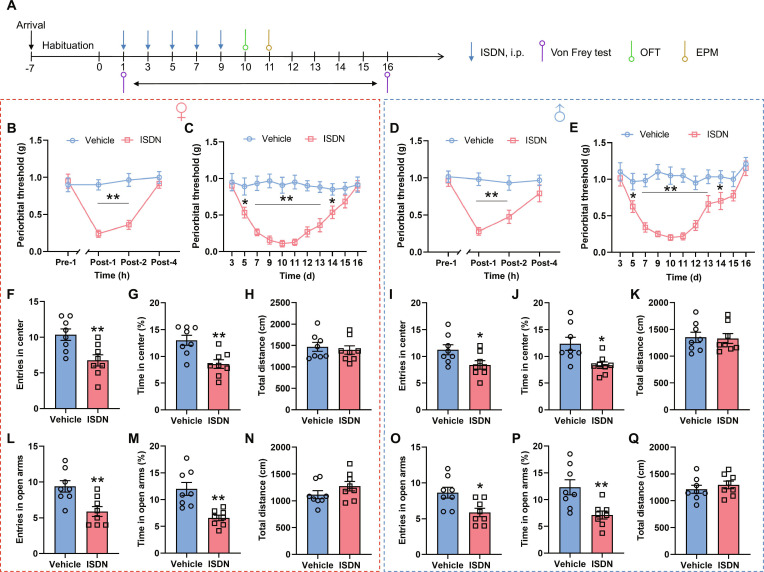
Repeated ISDN injections induce chronic headache with comorbid anxiety. (A) Experimental schedule for ISDN injections, Von Frey test, OFT, and EPM test. i.p., intraperitoneal. (B to E) Time course of single (B and D) and repeated (C and E) ISDN injections induced cephalic cutaneous allodynia measured with the Von Frey test in female (B and C) and male (D and E) mice. (F to K) Effects of repeated ISDN injections on the entries into enter zone (F and I), time spent in the center zone (G and J), and total distance traveled (H and K) of female (F to H) and male (I to K) mice in the OFT. (L to Q) Effects of repeated ISDN injection on the entries into open arms (L and O), time spent in the open arms (M and P), and total distance traveled (N and Q) of female (L to N) and male (O to Q) mice in the EPM. The data are presented as the mean ± SEM. *P<0.05, **P<0.01 versus Vehicle. Detailed statistical results are provided in Table S1.

The chronic headache mouse model was established with repeated intraperitoneal injections of the nitric oxide donor ISDN [32]. We employed the Von Frey test to measure cephalic cutaneous thresholds to mechanical stimuli at pre-1 h and post-1, -2, and -4 h of the first ISDN injection on day 1, immediately prior to the second to fifth ISDN injections on days 3, 5, 7, and 9, and once every day thereafter until day 16 (Fig. 1A). Noticeable cephalic cutaneous allodynia (indicator reflecting headache-like symptom) in male and female mice was detected at 1 and 2 h and disappeared at 4 h after the first ISDN injection, re-identified during days 5 to 14, and vanished on days 15 and 16 (Fig. 1B to E). On the 10th and 11th days after the establishment of chronic headache symptoms, anxiety-like behaviors were respectively detected through the open field test (OFT) and the elevated plus maze (EPM) test (Fig. 1A). Compared to the vehicle-injected control mice, the ISDN-injected male and female mice developed anxiety-like behaviors on days 10 and 11, characterized by marked reduction in entries into and the time spent in the central zone and open arms without apparent changes in total distance traveled (Fig. 1F to Q). These data demonstrate the establishment of repeated ISDN-induced headache and anxiety comorbidity in both male and female mice.

### Activation of AI, PrL, and ovBNST neurons

**Fig. 2.**
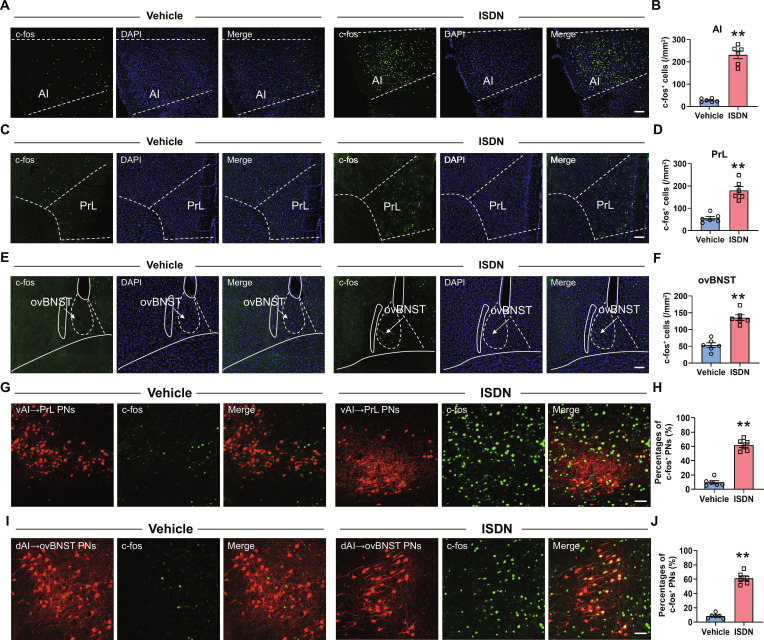
Repeated ISDN injections activate AI, PrL, and ovBNST neurons. (A to F) Representative images (A, C, and E) and graphs (B, D, and F) of c-fos-positive neurons in AI, PrL, and ovBNST after repeated ISDN or vehicle injections. Scale bar, 100 μm. (G and I) Representative images show c-fos expression in vAI→PrL projecting neurons (PNs) (G) and dAI→ovBNST PNs (I) of AI. AAV2/Retro-mCherry was injected into PrL (G) and ovBNST (I), resulting in retrograde expression in AI. Scale bar, 100 μm. (H and J) Statistical analysis shows the percentage of c-fos-positive neurons in vAI→PrL PNs (H) and dAI→ovBNST PNs (J) of AI. The data are presented as the mean ± SEM. **P<0.01 versus Vehicle. Detailed statistical results are provided in Table S1.

The association of AI, PrL, and ovBNST with pain and anxiety is well documented [28,33,34]. To investigate their involvement in headache and comorbid anxiety, c-fos immunofluorescent staining was conducted to identify activated neurons in AI, PrL, and ovBNST 2 h after the fifth vehicle or ISDN injection on day 9 [35]. Compared with vehicle-injected mice, ISDN-injected mice exhibited dramatically increased c-fos immunoreactivity in bilateral AIs, PrLs, and ovBNSTs (Fig. 2A to F), suggesting a potential association between their hyperactivity and comorbid headache and anxiety.

Moreover, immunostaining results showed robust c-fos expression in both PrL- and ovBNST-projecting AI neurons after ISDN injection (Fig. 2G to J), suggesting that activated PrL- and ovBNST-projecting AI neurons appeared to be involved in chronic headache and comorbid anxiety.

### Inhibition of AI circuits alleviates headache and anxiety

**Fig. 3.**
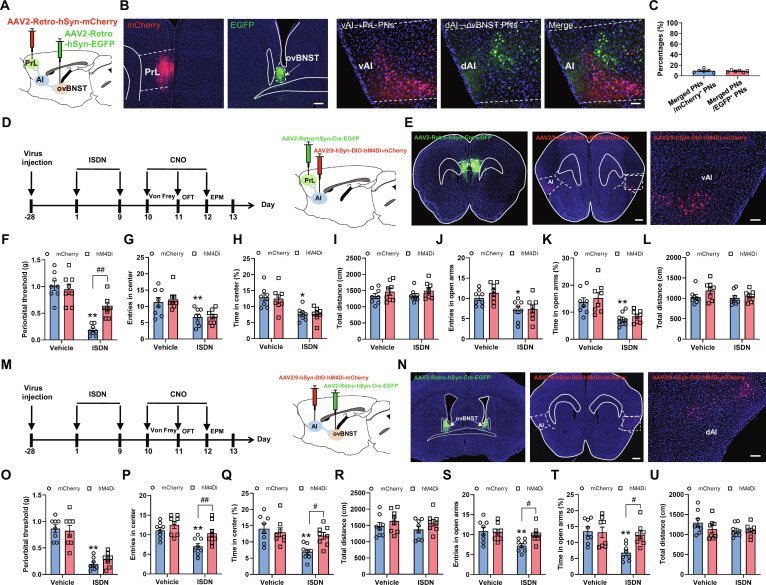
Chemo-inhibition of PrL- and ovBNST-projecting AI neurons ameliorates ISDN-induced headache and comorbid anxiety, respectively. (A) Schematic diagram for injections of mCherry- and EGFP-expressing retrogradely transported viruses into PrL and ovBNST, respectively. (B) Retrogradely transported viruses labeled PrL- and ovBNST-projecting neurons located in the ventral and dorsal parts of AI, respectively, with bare overlap. Scale bar, 200 μm (left) or 100 μm (right). (C) Statistical analysis shows the percentage of merged neurons in mCherry- or EGFP-expressing AI neurons. (D and M) Experimental schedule and schematic diagram for chemogenetic inhibition of PrL-projecting vAI neurons (D) and ovBNST-projecting dAI neurons (M). (E and N) Representative images show the EGFP-expressing neurons in the PrL (E) or ovBNST (N) and mCherry-expressing neurons in AI. Scale bars, 500 μm (left) or 100 μm (right). (F and O) Von Frey tests show effects of chemogenetic inhibition of PrL-projecting vAI neurons (F) and ovBNST-projecting dAI neurons (O) on cephalic cutaneous allodynia in mice with repeated ISDN injections. (G to L and P to U) OFT (G to I and P to R) and EPM test (J to L and S to U) show effects of chemogenetic inhibition of PrL-projecting vAI neurons (G to L) and ovBNST-projecting dAI neurons (P to U) on anxiety-like behaviors in mice with repeated ISDN injections. The data are presented as the mean ± SEM. *P<0.05, **P<0.01 versus Vehicle + mCherry. Detailed statistical results are provided in Table S1.

Given the robust neural connections between AI neurons and both PrL and ovBNST [29,36], we then used retrograde tracing adeno-associated viruses (AAVs) (Fig. 3A) to examine AI projection to PrL and ovBNST neurons. After injecting enhanced green fluorescent protein (EGFP)- or mCherry-expressing retrograde tracing viruses into PrL and ovBNST, we found that PrL and ovBNST neurons received direct innervation from 2 populations of AI neurons in layers II to IV, with ventrally located AI (vAI) neurons innervating PrL and dorsally located AI (dAI) neurons projecting to ovBNST (Fig. 3B and C).

Subsequently, we used a chemogenetic strategy to examine whether a selective inhibition of vAI-PrL and dAI-ovBNST circuits blocked ISDN induction of headache and anxiety by bilaterally injecting AAV2/retro-hSyn-Cre-EGFP into PrL or ovBNST and AAV2/9-hSyn-DIO-hM4Di-mCherry (AAV2/8-hSyn-DIO-mCherry was used as the control) into AI (Fig. 3D, E, M, and N). Whole-cell recordings in acute slices confirmed that clozapine N-oxide (CNO) effectively decreased the firing rates of hM4Di-expressing AI neurons (Fig. S1A to C), validating the efficacy of our chemogenetic inhibition strategy. Notably, behavioral assays revealed that in mice with ISDN-induced chronic headache and anxiety comorbidity, systemic CNO-evoked chemogenetic inhibition of PrL-projecting vAI neurons attenuated cephalic cutaneous allodynia (Fig. 3F) without altering anxiety-like behaviors in the OFT (Fig. 3G to I) or EPM (Fig. 3J to L) tests. Conversely, inhibition of ovBNST-projecting dAI neurons abolished ISDN-induced anxiety-like behaviors (Fig. 3P to U) while leaving cephalic cutaneous allodynia unaffected (Fig. 3O). Collectively, these findings indicate that suppressing the vAI-PrL and dAI-ovBNST circuits differentially prevents ISDN-triggered chronic headache and anxiety-like behaviors.

### Headache and anxiety enhance eCB release in AI circuits

**Fig. 4.**
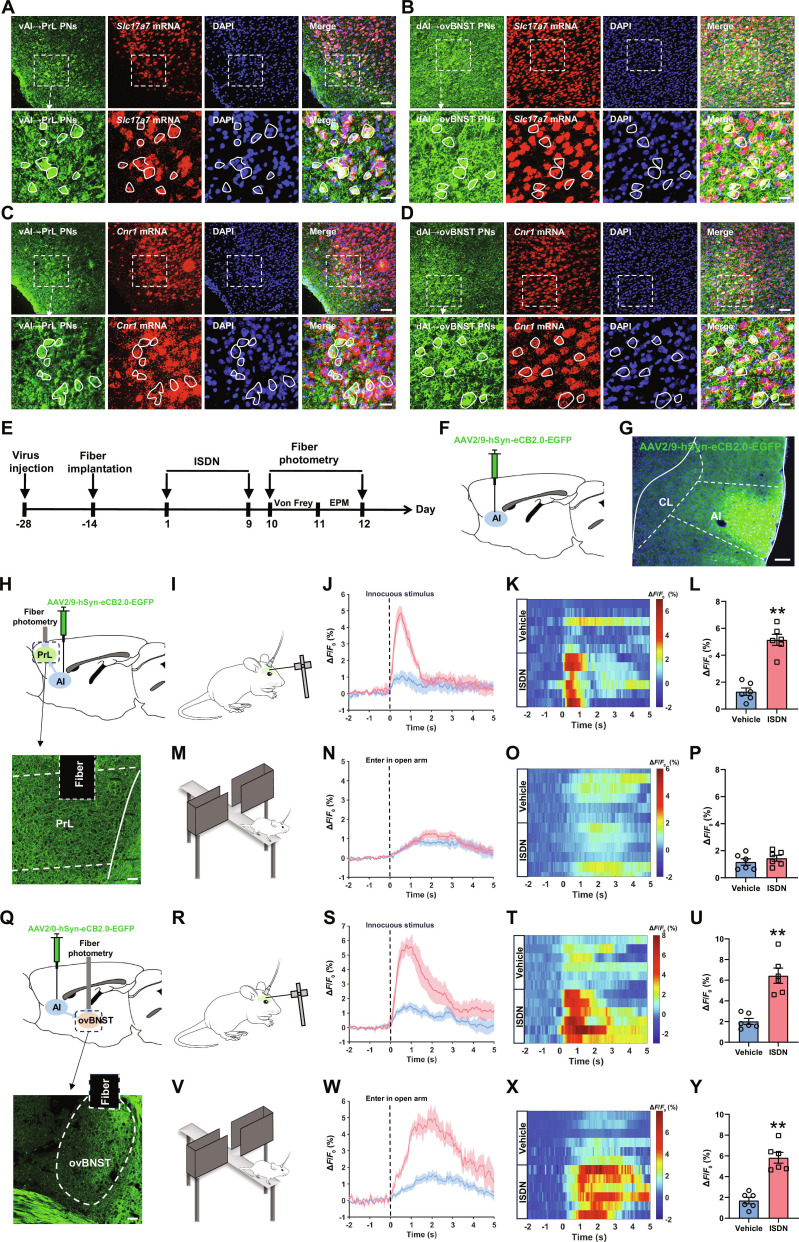
Repeated ISDN injections potentiate eCB release in vAI-PrL and dAI-ovBNST synapses during Von Frey and EPM tests. (A to D) RNAscope and immunofluorescence costaining show the expression of *Cnr1* mRNA (the mRNA of CB1R) (A and B) and *Slc17a7* mRNA (a marker of glutamatergic neurons) (C and D) in PrL-projecting dAI neurons (A and C) and ovBNST-projecting vAI neurons (B and D). Scale bars, 100 μm (top) or 20 μm (bottom). (E to H and Q) Experimental schedule (E) for viral injections into AI (F and G) with fiber implantation and eCB signal recording above PrL (H) or ovBNST (Q). Representative images show eCB2.0 sensor-expressing neurons in the AI (G) or eCB2.0 sensor-expressing projections in PrL (H) or ovBNST (Q). Scale bar, 200 μm. (I to P and R to Y) Dynamic trace changes (J, N, S, and W), heatmaps (K, O, T, and X), and graphs (L, P, U, and Y) of eCB signals in vAI-PrL and dAI-ovBNST synapses during Von Frey (I to L and R to U) and EPM (M to P and V to Y) tests in mice with repeated vehicle or ISDN injections. The data are presented as the mean ± SEM. **P<0.01 versus Vehicle. Detailed statistical results are provided in Table S1.

eCBs released from the postsynaptic membrane modulate diverse neural functions, including pain perception and emotional states, via retrograde activation of the CB1R [7,9]. Given that our RNAscope analysis revealed dense *Cnr1* mRNA expression (encoding CB1R) in both PrL-projecting and ovBNST-projecting AI glutamatergic neurons (Fig. 4A to D), it is possible that eCB signaling within the vAI-PrL and dAI-ovBNST circuits regulates the comorbidity of headache and anxiety. To test this hypothesis, we employed the projection- and synapse-specific eCB2.0 sensor [37] to directly examine eCB release at vAI-PrL and dAI-ovBNST synapses in mice during cephalic cutaneous allodynia and anxiety in chronic ISDN-injected mice. We injected AAV2/9-hSyn-eCB2.0-EGFP into the unilateral AI and then implanted optical fibers above the ipsilateral PrL or ovBNST (Fig. 4E to G, H, and Q). Confined EGFP-expressing cells in the AI (Fig. 4G) and EGFP-expressing projections in PrL (Fig. 4H) and ovBNST (Fig. 4Q) were exhibited in virus-injected mice, confirming the accuracy of the virus injection. Real-time synaptic eCB dynamics were recorded during the Von Frey and EPM tests in vehicle- and ISDN-injected mice (Fig. 4I, M, R, and V). In comparison with vehicle-injected mice, ISDN-injected mice showed a remarkable elevation of eCB release of vAI neuronal projections in PrL during the Von Frey test (Fig. 4J to L) but not in the EPM test (Fig. 4N to P), suggesting that the eCB of vAI-PrL circuit is likely involved in headache rather than anxiety. The eCB releases of dAI-ovBNST synapse were also strikingly increased during both Von Frey test (Fig. 4S to U) and EPM test (Fig. 4W to Y), suggesting that the eCB of dAI-ovBNST circuit is likely involved in both headache and anxiety comorbidity.

### Insignificant effects of eCB reduction in AI circuits on headache and anxiety

**Fig. 5.**
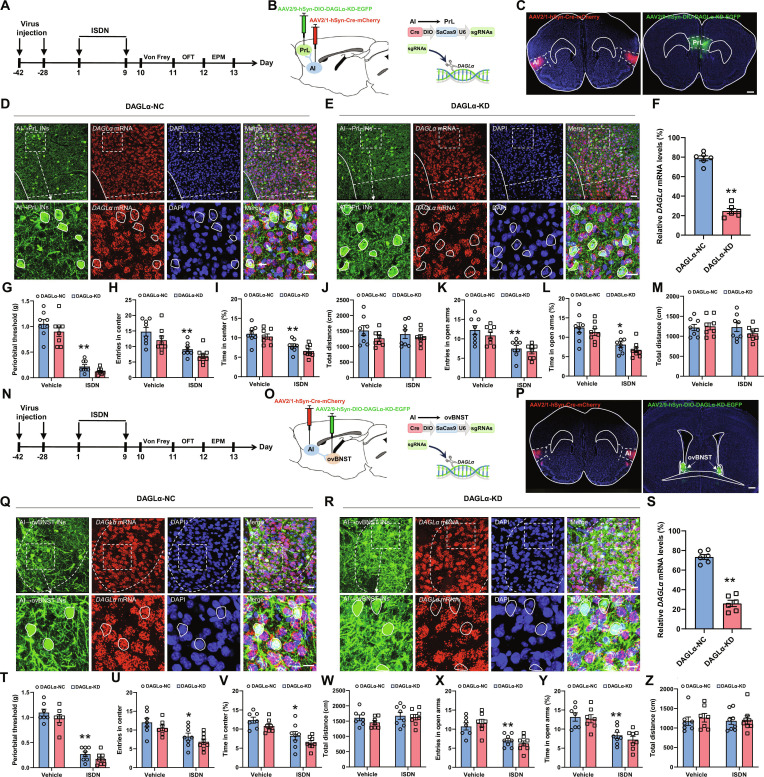
Knockdown of DAGLα in vAI-PrL and dAI-ovBNST synapses has no effect on cephalic cutaneous allodynia and anxiety-like behaviors in ISDN-injected mice. (A to C and N to P) Experimental schedule for DAGLα knockdown in vAI-PrL (A and B) and dAI-ovBNST synapses (N and O), viral injections, and behavioral tests. Representative images show mCherry-expressing neurons in the AI (C and P) and EGFP-expressing neurons in PrL (C) and ovBNST (P). Scale bars, 500 μm. (D to F and Q to S) RNAscope and immunofluorescent costaining show the effective knockdown of the expression of *DAGLα* mRNA in AI-innervating PrL (D to F) and ovBNST (Q to S) neurons. Dotted coils indicate neurons infected with AAV viruses. Scale bars, 100 μm (top) or 20 μm (bottom). (G and T) Von Frey tests show effects of DAGLα-KD in vAI-PrL (G) or dAI-ovBNST (T) on repeated ISDN-induced cephalic cutaneous allodynia. (H to M and U to Z) OFT (H to J and U to W) and EPM tests (K to M and X to Z) show the effects of DAGLα-KD in vAI-PrL (H to M) or dAI-ovBNST (U to Z) on repeated ISDN-induced anxiety-like behaviors. DAGLα-NC, DAGLα noncoding control; DAGLα-KD, DAGLα knockdown; INs: innervating neurons. The data are presented as the mean ± SEM. **P<0.01 versus DAGLα-NC (F and S). *P<0.05, **P<0.01 versus Vehicle + DAGLα-NC (G to M and T to Z). Detailed statistical results are provided in Table S1.

Given that eCB levels are elevated at activated synapses in the vAI-PrL and dAI-ovBNST circuits during behavioral states of headache and anxiety, we logically inferred that reducing eCB levels specifically at these synapses should ameliorate cephalic cutaneous allodynia and anxiety-like behaviors induced by ISDN injection. To this end, we employed a genetic strategy to knock down the 2-AG synthase DAGLα in a circuit-specific manner [8,12]. We delivered AAV2/1-hSyn-Cre-mCherry into bilateral AI, followed 2 weeks later by the injections of a mixture of AAV2/9-CMV-EGFP-WPRE-U6-DAGLα-sgRNAs and AAV2/9-hSyn-DIO-SaCas9-3xFLAG (1:1) (AAV2/9-hSyn-DIO-DAGLα-KD-EGFP) into bilateral PrLs or ovBNSTs. Four weeks later, we performed repeated ISDN injections and then conducted behavioral tests (Fig. 5A to C and N to P). Our RNAscope results revealed a marked reduction in DAGLα expression levels within neurons transduced with DAGLα-knockdown (KD) viruses, compared to control virus-infected neurons (Fig. 5D to F and Q to S). This quantitative evidence confirmed the successful suppression of *DAGLα* mRNA expression specifically in PrL- and ovBNST-projecting AI neurons, respectively. Surprisingly, behavioral testing revealed that knockdown of DAGLα in either the vAI-PrL or dAI-ovBNST circuit ‌failed to block cephalic cutaneous allodynia or anxiety-like behavior in ISDN-injected mice (Fig. 5G to M and T to Z), suggesting that ‌reducing eCB levels‌ within vAI-PrL and dAI-ovBNST synapses ‌does not affect‌ headache or comorbid anxiety. Collectively, these results indicate that in the comorbid headache and anxiety model, increased eCB release within the vAI-PrL and dAI-ovBNST circuits during Von Frey or EPM exposure does not initiate cephalic cutaneous allodynia or anxiety-like behaviors.

### eCB elevation in AI circuits attenuates headache and anxiety

**Fig. 6.**
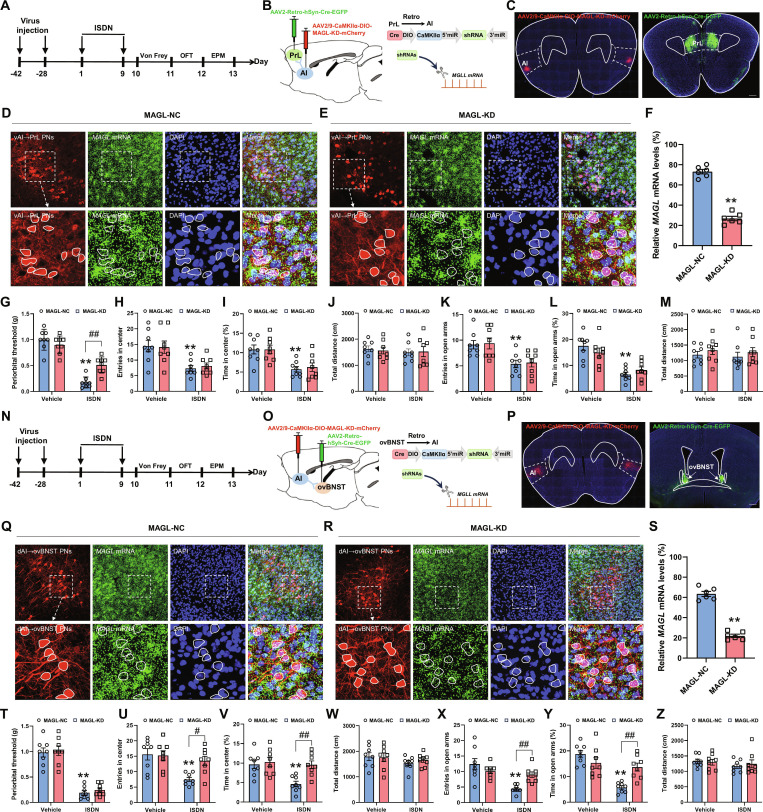
Knocking down MAGL in vAI-PrL and dAI-ovBNST synapses ameliorates headache and comorbid anxiety, respectively. (A to C and N to P) Experimental schedule for MAGL knockdown in vAI-PrL (A and B) and dAI-ovBNST (N and O) synapses, viral injections, and behavioral tests. Representative images show mCherry-expressing neurons in the AI (C and P) and EGFP-expressing neurons in PrL (C) and ovBNST (P). Scale bars, 500 μm. (D to F and Q to S) RNAscope and immunofluorescence costaining show the effective knockdown of the expression of *MAGL* mRNA in vAI-PrL (D to F) and dAI-ovBNST (Q to S) circuits. Dotted coils indicate neurons infected with AAV viruses. Scale bars, 100 μm (top) or 20 μm (bottom). (G and T) Von Frey tests show effects of MAGL-KD in vAI-PrL (G) or dAI-ovBNST (T) on cephalic cutaneous allodynia. (H to M and U to Z) OFT (H to J and U to W) and EPM tests (K to M and X to Z) show effects of MAGL-KD in vAI-PrL (H to M) or dAI-ovBNST (U to Z) on anxiety-like behaviors. MAGL-NC, MAGL noncoding control; MAGL-KD, MAGL knockdown. The data are presented as the mean ± SEM. **P<0.01 versus MAGL-NC (F and S). *P<0.05, **P<0.01 versus Vehicle + MAGL-NC (G to M and T to Z). Detailed statistical results are provided in Table S1.

Since the knockout of eCB in both vAI-PrL and dAI-ovBNST synapses has no effect on alleviating headache and anxiety-like behaviors, we next examined whether enhancing eCB signaling could block these behaviors. To achieve this, we employed a well-established circuit-specific silencing approach targeting *MAGL* mRNA to selectively increase 2-AG levels at vAI-PrL and dAI-ovBNST synaptic clefts [12]. Thus, AAV2/retro-hSyn-Cre-EGFP were injected into bilateral PrL or ovBNST, followed 2 weeks later by injection of AAV2/9-CaMKII-DIO-mCherry-miR30shRNA (MAGL)-WPRE [or AAV2/9-CaMKII-DIO-mCherry-miR30shRNA (NC)-WPRE as control] into bilateral AI and another 4 weeks later by repeated ISDN injections and subsequent behavioral tests (Fig. 6A to C and N to P). RNAscope results confirmed a dramatical reduction of MAGL expression levels within neurons infected with MAGL-KD viruses, compared to control virus-infected neurons (Fig. 6D to F and Q to S), suggesting the successful suppression of *MAGL* mRNA expression specifically in PrL- and ovBNST-projecting AI neurons, respectively. Behaviorally, the vehicle-injected mice receiving MAGL knockdown exhibited no statistically significant alterations in Von Frey, OFT, and EPM tests (Fig. 6G to M and T to Z), suggesting that the increase of eCB level in vAI-PrL and dAI-ovBNST synapses has no obvious effect on nociception and affective behaviors in control mice. Notably, the ISDN-injected mice with specific knockdown of MAGL in PrL-projecting vAI neurons showed a remarkable improvement of cephalic cutaneous allodynia (Fig. 6G) without affecting the anxiety-like behaviors (Fig. 6H to M). In contrast, MAGL knockdown in ovBNST-projecting dAI neurons blocked anxiety-like behaviors of ISDN-injected mice (Fig. 6U to Z) while exhibiting no obvious effect on cephalic cutaneous allodynia (Fig. 6T). These results suggested that enhancing eCB in vAI-PrL and dAI-ovBNST synapses alleviated headache and anxiety, respectively.

### CB1R activation in AI circuits alleviates headache and anxiety

**Fig. 7.**
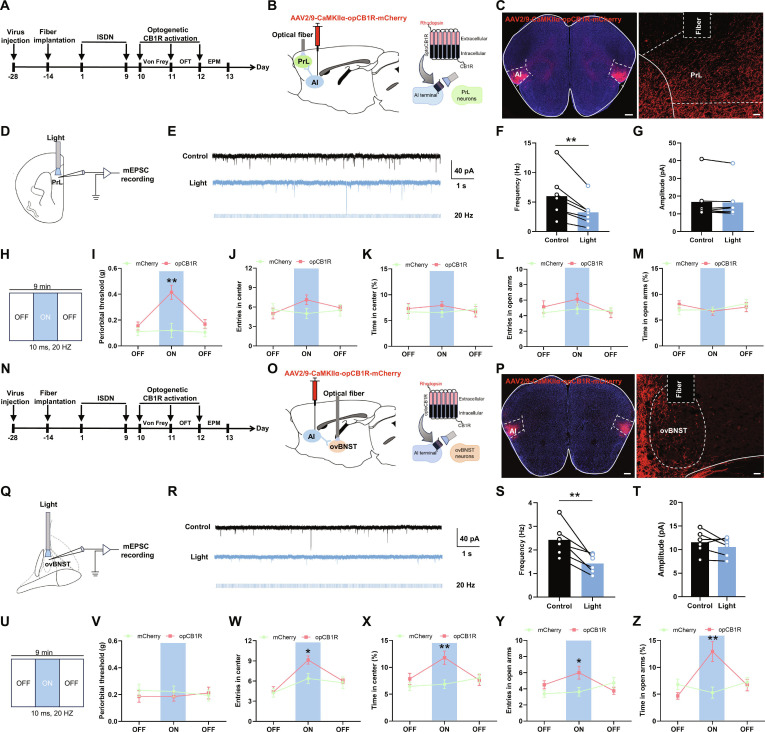
Optogenetic activation of CB1R in vAI-PrL and dAI-ovBNST synapses ameliorates cephalic cutaneous allodynia and anxiety-like behaviors in ISDN-injected mice. (A to C, H, N to P, and U) Experimental schedule for optogenetic activation of CB1R in vAI-PrL (A, B, and H) and dAI-ovBNST (N, O, and U) synapses, optical fiber implantation, viral injections, and behavioral tests. Representative images show opCB1R-expressing neurons in the AI (C and P) and opCB1R-expressing projections in PrL (C) or ovBNST (P). Scale bars, 500 μm (left) or 100 μm (right). (D to G and Q to T) Schematic diagram (D and G), representative raw trace (E and R), and quantitative frequency (F and S) and amplitude (G and T) of miniature excitatory postsynaptic current (mEPSC) recording in PrL (D to G) and ovBNST (Q to T). (I to M and V to Z) Effects of optogenetic activation of CB1R in vAI-PrL (I to M) and dAI-ovBNST (V to Z) synapses on repeated ISDN-induced cephalic cutaneous allodynia in Von Frey test (I and V) and anxiety-like behaviors in the OFT (J, K, W, and X) and EPM tests (L, M, Y, and Z). The data are presented as the mean ± SEM. *P<0.05, **P<0.01 versus Control or mCherry. Detailed statistical results are provided in Table S1.

Given that synaptic eCBs retrogradely activate presynaptic CB1Rs to suppress presynaptic neurotransmitter release, it was essential to examine whether eCB-mediated analgesia and anxiolysis in vAI-PrL and dAI-ovBNST synapses depend on presynaptic CB1R activation. To this end, we employed the recently developed opCB1R strategy to precisely activate CB1Rs in a circuit- and synapse-specific manner by expressing a photosensitive rhodopsin-modified CB1R (opCB1R) in AI somata and terminals [8,12]. AAV2/9-CaMKII-opCB1R-mCherry was injected into bilateral AI, and optical fibers were implanted into bilateral PrL or ovBNST, which was followed by performing ISDN injection, opto-stimulation, and subsequent behavioral tests (Fig. 7A, B, N, and O). Viral tracing in bilateral AI revealed robust mCherry-labeled AI neurons with clearly identifiable axonal projections terminating in PrLs (Fig. 7C) and ovBNSTs (Fig. 7P). To verify the synaptic regulation of opCB1Rs in the vAI-PrL and dAI-ovBNST circuits, we recorded the miniature excitatory postsynaptic currents (mEPSCs) in areas of high opCB1R-mCherry terminal infectivity in PrL and ovBNST with blue light (473 nm, 20 Hz, 10-ms pulses) (Fig. 7D and Q). Our results revealed that blue light stimulation vividly decreased the frequency of mEPSC without affecting the amplitude, suggesting that the activation of opCB1R reduced the glutamate release in these synapses (Fig. 7E to G and R to T). Moreover, light stimulation of opCB1Rs in vAI-PrL projections evoked a remarkable alleviation of cephalic cutaneous allodynia (Fig. 7H and I) without affecting anxiety-like behavior (Fig. 7J to M). Conversely, activation of opCB1Rs in dAI-ovBNST projections abolished anxiety-like behaviors (Fig. 7U and W to Z) without influencing cephalic cutaneous allodynia (Fig. 7V). These results indicated that selective activation of presynaptic CB1Rs in vAI-PrL and dAI-ovBNST synapses ameliorated the ISDN-induced headache and anxiety-like behaviors, respectively.

### CB1R antagonism reverses JZL184-induced analgesia and anxiolysis

**Fig. 8.**
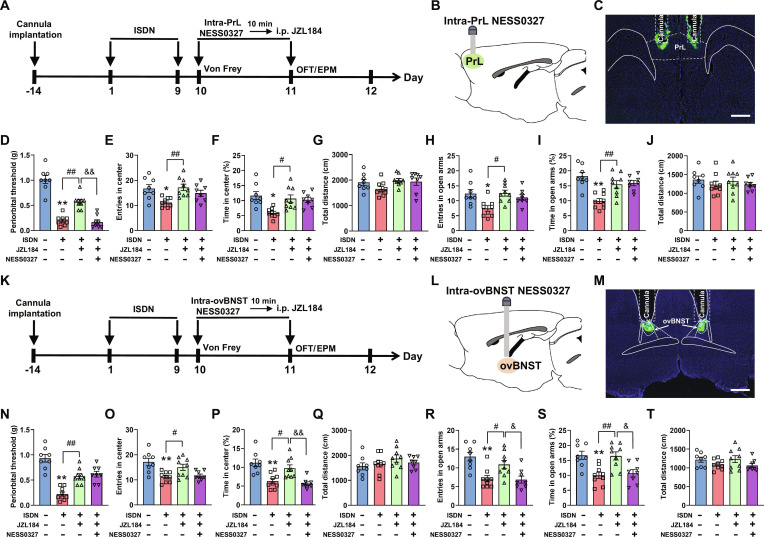
CB1R antagonism in PrL- and ovBNST-reversed JZL184 produced analgesic and anxiolytic effects in mice with repeated ISDN injections. (A to C and K to M) Experimental schedule (A and K) and schematic diagram (B and L) for bilateral cannula implantation above bilateral PrLs or ovBNSTs, local injection of the CB1R antagonist NESS0327 into bilateral PrLs (B and C) or ovBNSTs (L and M), and intraperitoneal injection of 2-AG hydrolase (MAGL) inhibitor JZL184. Representative images show cannula tracks above PrLs (C) and ovBNSTs (M). The green fluorescent CTB-488 dye enables visualization of injection sites. Scale bars, 200 μm. (D to J and N to T) Effects of intra-PrL (D to J) or intra-ovBNST injection (N to T) of NESS0327 (0.5 μM/300 nl/side) and systematic administration of JZL184 (10 mg/kg) on repeated ISDN-induced cephalic cutaneous allodynia in Von Frey test (D and N) or anxiety-like behaviors in the OFT (E to G and O to Q) and EPM tests (H to J and R to T). The data are presented as the mean ± SEM. *P<0.05, **P<0.01 versus Vehicle group. Detailed statistical results are provided in Table S1.

The results described above indicate that enhanced eCB signaling in the vAI-PrL and dAI-ovBNST circuits ameliorates headache and anxiety, respectively. Therefore, it is reasonable to hypothesize that systemic augmentation of eCB signaling should simultaneously prevent both cephalic cutaneous allodynia and anxiety-like behavior in ISDN-injected mice. To directly test this hypothesis, we intraperitoneally injected the MAGL inhibitor JZL184, at a dosage of 10 mg/kg, 2 h before the behavioral tests. As expected, the ISDN-injected mice with administration of JZL184 showed marked improvement in both cephalic cutaneous allodynia and anxiety-like behaviors (Fig. 8D to J and N to T), suggesting that systemic augmentation of eCB signaling can simultaneously improve headache and anxiety. To further validate the critical role of eCB in the vAI-PrL and dAI-ovBNST circuits for modulating headache and comorbid anxiety, we combined systemic intraperitoneal administration of JZL184 with local microinjections of the CB1R antagonist NESS0327 into PrL and ovBNST, respectively, followed by behavioral assessment in mice (Fig. 8A to C and K to M). Our behavioral results demonstrated that bilateral microinjections of NESS0327 (0.5 μM, 300 nl/side) into PrL and ovBNST counteracted JZL184’s analgesic and anxiolytic effects, respectively (Fig. 8D to J and N to T). These findings indicate that enhancing eCB in the vAI-PrL and dAI-ovBNST circuits is essential for relieving headache and anxiety, respectively.

## Discussion

**Fig. 9.**
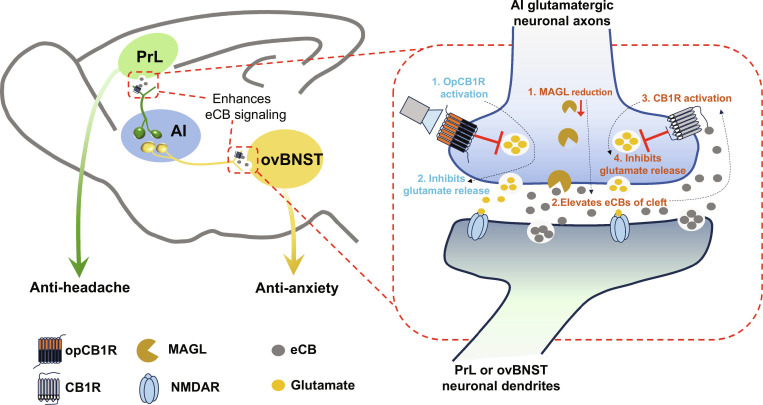
Summary of eCB function enhancement in vAI-PrL and dAI-ovBNST circuits to improve headache and comorbid anxiety. Repeated ISDN injections pathologically activated 2 spatially segregated glutamatergic neuronal populations in AI, with one situated in its ventral region innervating PrL neurons, and the other located in its dorsal region output to ovBNST neurons. Hyperactivation of vAI-PrL and dAI-ovBNST circuits drives headache and anxiety phenotypes, respectively, while simultaneously inducing an adaptive response, up-regulating eCB release within these pathways in an attempt to attenuate neuronal hyperexcitability (a compensatory mechanism that was ineffective). Thus, chemogenetic inhibition of vAI-PrL and dAI-ovBNST circuits or targeted potentiation of their eCB signaling (opCB1R activation or MAGL reduction) blocked the cephalic cutaneous allodynia and anxiety-like behaviors, respectively. AI, anterior insular cortex; ovBNST, oval subdivision of the bed nucleus of the stria terminalis; PrL, prelimbic cortex; CB1R, cannabinoid type 1 receptor; opCB1R, photosensitive rhodopsin-modified CB1R; MAGL, monoacylglycerol lipase; eCB, endocannabinoid.

In primary headache disorders, anxiety represents the most prevalent psychiatric comorbidity, affecting up to 52% of patients globally [1,2]. However, the precise pathogenesis underlying this comorbidity remains elusive. This challenging clinical landscape necessitates urgent development of dual-target therapies for chronic headache and comorbid anxiety. In this study, we integrated neuroanatomical tracing, in vivo imaging, behavioral assessment, chemogenetic approaches, and 4 innovative synapse-specific eCB-targeted viral strategies in a validated chronic headache model to demonstrate the divergent role of inhibition of vAI-PrL and dAI-ovBNST circuits in headache and anxiety. Our study revealed that repeated ISDN administration pathologically activates 2 spatially segregated populations of glutamatergic neurons within AI: a ventral subpopulation innervating PrL neurons and a dorsal subpopulation projecting to ovBNST neurons. Hyperactivation of the vAI-PrL and dAI-ovBNST neural circuits drives headache and anxiety phenotypes, respectively, while simultaneously inducing an adaptive response that up-regulates eCB release within these pathways. This compensatory mechanism attempts to reduce neuronal hyperexcitability—though ultimately proving ineffective. Consequently, chemogenetic inhibition of vAI-PrL/dAI-ovBNST circuits or targeted potentiation of their eCB signaling abolishes cephalic cutaneous allodynia and anxiety-like behaviors, respectively (Fig. 9). Critically, systemic administration of eCB degradation inhibitors simultaneously ‌rescued‌ chronic ISDN-induced headache and anxiety comorbidity, which are separately abolished by CB1R antagonist application in PrLs and ovBNSTs, demonstrating that eCB potentiation in the vAI-PrL and dAI-ovBNST circuits is indispensable for alleviating headache and anxiety, respectively. Collectively, our findings reveal divergent counteracting effects of elevated eCB signaling in vAI-PrL and dAI-ovBNST circuits on comorbid headache and anxiety.

AI is a crucial brain region responsible for modulating nociceptive and emotional information, and its impairment was associated with hyperalgesia and heightened anxiety [27,38–40]. However, it is entirely unknown whether and how AI neurons regulate the pathophysiology of headache and anxiety comorbidity. In this study on the mouse model of chronic headache and anxiety comorbidity after ISDN injections, we observed intensive c-fos expression in AI neurons and their downstream PrL and ovBNST, indicating their involvement in comorbid headache and anxiety. We discovered that the mouse AI has 2 nonoverlapping subpopulations of neurons, with vAI neurons innervating the PrL and dAI neurons innervating ovBNST. Chemogenetic inhibition of vAI-PrL and dAI-ovBNST circuits respectively blocked ISDN-induced headache and anxiety, indicating the critical role of vAI-PrL and dAI-ovBNST circuits in inducing headache and anxiety, respectively. Collectively, our study identified 2 anatomically segregated neuronal populations within AI that differentially modulate headache and comorbid anxiety via distinct projections to PrL and ovBNST, respectively.

eCBs are synthesized under the activation state of postsynaptic neurons to suppress presynaptic neurotransmitter release, thereby regulating synaptic plasticity and maintaining neurophysiological homeostasis [7]. Clinically, patients with eCB deficiency syndrome demonstrate heightened migraine and headache susceptibility, indicating pathophysiological involvement of eCB signaling in headache disorders [41]. However, whether and how eCBs could serve as a unified therapeutic approach for headache–anxiety comorbidity remains unclear. In this study, utilizing the eCB2.0 biosensor, we detected strikingly elevated eCB release within vAI-PrL synaptic clefts during cephalic cutaneous mechanical stimuli under chronic headache conditions, whereas no difference was observed versus controls during anxiety-like behavioral assays. Conversely, dAI-ovBNST circuits exhibited increased eCB release during both headache and anxiety testing, suggesting that eCB signaling in vAI-PrL and dAI-ovBNST circuits is involved in the regulation of headache–anxiety comorbidity. Surprisingly, circuit-specific DAGLα knockdown to reduce synaptic eCB release in vAI-PrL and dAI-ovBNST pathways failed to alleviate headache-like cephalic cutaneous allodynia or anxiety-like behaviors. In contrast, circuit-specific MAGL knockdown to elevate eCB or optogenetic CB1R activation in these pathways prevented cephalic cutaneous allodynia and anxiety-like behaviors, respectively. These results suggest that the elevated eCB release observed in both vAI-PrL and dAI-ovBNST circuits likely constitutes a compensatory mechanism—an endogenous attempt to restore synaptic homeostasis through retrograde suppression of presynaptic glutamate release—in response to pathological hyperactivation of these neural pathways. However, this compensatory response appears insufficient to fully counteract the neural hyperactivity driving the pathological states. The underlying reason may be that endogenous eCB production capacity is fundamentally limited compared to the sustained hyperactivity in these circuits, resulting in eCB release below the threshold required for therapeutic benefit. Collectively, these findings reveal that in the headache–anxiety comorbidity model, increased eCB release within the vAI-PrL and dAI-ovBNST circuits during Von Frey or EPM exposure does not account for the occurrence of cephalic cutaneous allodynia or anxiety-like behaviors. Rather, this neuromodulatory response likely represents a compensatory adaptation attempting to dampen hyperexcitable circuits and prevent nociceptive and affective symptoms—though ultimately proving ineffective.

‌Interestingly, chemogenetic inhibition of dAI-ovBNST circuits markedly improved anxiety-like behaviors without affecting cephalic cutaneous allodynia. Enhancing dAI-ovBNST synaptic eCB or activating CB1R similarly blocked anxiety-like behaviors without altering nociceptive sensitization. These results demonstrate that dAI-ovBNST circuitry does not participate in headache regulation. Nevertheless, eCB2.0 biosensor recordings revealed marked eCB release in dAI-ovBNST synapses during Von Frey testing. We postulate that Von Frey testing, which was employed to measure cephalic cutaneous allodynia in chronic ISDN-injected mice, may elicit pain sufficient to function as a potent anxiogenic stressor, thereby recruiting dAI-ovBNST projections.

eCB activation has been demonstrated to ameliorate various pathologies including pain and anxiety [42–45]. Our study revealed that systematic administration of JZL184 elicited potent analgesic and anxiolytic effects in a mouse model with headache and anxiety comorbidity, indicating that eCB system activation may serve as a unified therapeutic strategy for simultaneously treating cephalalgia and comorbid anxiety. Moreover, localized CB1 receptor antagonism in PrL and ovBNST respectively reversed JZL184’s analgesic and anxiolytic effects, further establishing the indispensable, differential roles of vAI-PrL and dAI-ovBNST circuits in regulating cephalalgic hyperalgesia and anxiety-like behaviors.

Notably‌, while the ISDN-induced mouse model is one of the most widely used for migraine research due to its high similarity to human migraine symptoms, the complex and multifactorial pathogenesis of migraine cannot be fully replicated by mere vascular dilation. The ISDN-triggered allodynia is transient and reversible, whereas chronic migraine-associated allodynia typically persists for months or longer, suggesting distinct underlying pathological mechanisms. Therefore, this study primarily focuses on interventions for ISDN-induced allodynia rather than chronic migraine-related symptoms.

In conclusion, this study provides a comprehensive analysis of the vAI-PrL and dAI-ovBNST circuits, elucidating the function of eCB signaling within these pathways in modulating headache–anxiety comorbidity. Specifically, we demonstrate for the first time that chemogenetic inhibition of vAI-PrL and dAI-ovBNST circuits versus targeted enhancement of eCB signaling within these pathways divergently ameliorated headache and anxiety. These findings thereby establish eCB neuromodulation as a clinically actionable unitary therapeutic target for the concurrent treatment of comorbid headache and anxiety disorders.

## Materials and Methods

### Animal

Male and female adult C57BL/6J mice (8 to 12 weeks, 20 to 25 g, Beijing Vital River Laboratory Animal Technology Co. Ltd.) were group-housed in 2 to 4 per cage and maintained in a reversed 12-h light/dark cycle (room temperature 22 to 26 °C, humidity around 50%). The mice were given free access to food and water. All experimental procedures were carried out strictly in accordance with the regulations of the institution and were approved by the Animal Care Committee of Qingdao University.

### Chronic headache model establishment

The chronic headache mouse model was created by multiple nitric oxide donor intraperitoneal injections according to previous literature [46]. Specifically, ISDN (S28294, Yuanye Bio-Technology, Shanghai, China) was prepared by dissolving 10 mg in 100 μl of dimethyl sulfoxide (DMSO; Sigma-Aldrich, USA), followed by dilution with 0.9% saline to achieve a final concentration of 1 mg/ml. The mice were given an intraperitoneal injection of ISDN (at a dose of 10 mg/kg) or the same volume vehicle control (1% DMSO in saline) every other day for 9 consecutive days (a total of 5 injections).

### Behavioral assessments

The behavioral assessments were conducted in a quiet and dim (20 lux) environment during the light cycle (10:00 AM to 4:00 PM). Mice were allowed 30 min of acclimation to the testing environment before behavioral tests. According to previous studies [47,48], cephalic cutaneous allodynia was assessed using the Von Frey test, and anxiety-like behaviors were evaluated using the open field and EPM tests.

### Von Frey test

Given that cephalic cutaneous allodynia is a hallmark clinical manifestation in chronic headache patients [49], we employed Von Frey filaments to assess mechanical pain thresholds in mice, focusing on the trigeminal nerve-innervating region (cephalic cutaneous area). Mice were habituated to handling and innocuous mechanical stimulation for at least 5 d prior to the periorbital threshold measurements. Animals with a baseline threshold below 0.16 g were excluded. Periorbital thresholds were measured using Von Frey filaments (Stoelting, IL, USA; range: 0.02 to 2 g) via the up-down method [50,51]. Frey filaments (starting at 0.16 g) were applied to the cephalic cutaneous skin in a vertical direction for 3 s with slight bending, and a rapid head withdrawal or scratching was considered a positive response. Three trials were conducted with 5-min intervals between each one. Then, the average value was calculated as the periorbital threshold.

### Open field test

For the OFT, a white plastic box (45 cm × 45 cm × 30 cm) was placed under a high-resolution video camera. During behavioral testing, mice were carefully positioned in the central zone (15 cm × 15 cm) of the apparatus and permitted unrestricted exploration for a standardized 6-min observation period. To control for olfactory cues, the testing chamber was systematically sanitized with 75% ethanol solution between trials, followed by a mandatory 3-min drying interval to ensure complete odor dissipation before subsequent testing. The movement trajectory of each mouse was analyzed by EthoVision XT software (Noldus Information Technology, Netherlands). Anxiety-like behavior was assessed by counting the entries into and the time spent in the central zone and the total distance traveled during the test.

### Elevated plus maze

The EPM apparatus, positioned 50 cm above ground level, consisted of a central quadrangular platform (5 cm × 5 cm) serving as the behavioral transition zone, 2 mutually perpendicular open arms (30 cm × 5 cm × 0.5 cm) with raised edge rails (0.5 cm height), and 2 enclosed arms (30 cm × 5 cm × 15 cm) featuring opaque polycarbonate walls. For standardized behavioral assessment, mice were individually introduced to the EPM with precise initial orientation. Between trials, the apparatus underwent rigorous decontamination using a 75% ethanol solution, applied uniformly across all maze surfaces, followed by complete evaporation (≥3 min) to eliminate residual olfactory cues that might influence subsequent behavioral measurements. Movement trajectories were analyzed using the EthoVision XT software (Noldus Information Technology, Netherlands) to track the entire behavioral sequence. Anxiety-like behavior was assessed by counting the number of entries into the open arms, the time spent in the open arms, and the total distance traveled during the test.

### Stereotaxic AAV injection

Mice were fixed in a stereotactic frame (Kopf Instruments) under an intraperitoneal injection of 50 mg/kg of 1% pentobarbital. The viral injections were conducted using the pulled glass microelectrodes (GC-3.5, RWD Life Science, Shenzhen, China) connected with a 10-μl syringe. A volume of 150 nl of virus was injected into the target sites using an infusion pump (LEGATO 130, Kd Scientific), which delivered the virus at a constant rate of 30 nl/min. The stereotactic injection coordinates were defined based on The Mouse Brain in Stereotaxic Coordinates (Paxinos and Franklin’s, 2nd edition). The coordinates were identified as the following 3 indicators: dorsal–ventral (DV) from the brain surface, anterior–posterior (AP) from bregma, and medial–lateral (ML) from the midline. The target sites and the corresponding coordinates (AP, ML, DV) were listed as follows: AI (+1.80 mm, ±3.00 mm, −2.70 mm), PrL (+1.80 mm, ±0.30 mm, −2.30 mm), and ovBNST (+0.30 mm, ±0.90 mm, −3.90 mm).

For the monosynaptic retrograde tracing experiments, we microinjected PrL or ovBNST with AAV2/retro-hSyn-mCherry (AAV2-Retro, titer: 5.60 × 10^12^ vg/ml, OBiO, China) or AAV2/retro-hSyn-EGFP (AAV2-Retro, titer: 2.16 × 10^13^ vg/ml, OBiO, China), respectively. Following a 2-week recovery period, the mice were sacrificed to determine the distribution of AI neurons projecting to PrL and ovBNST.

For chemogenetic inhibition of AI neurons projecting to PrL or ovBNST, we bilaterally microinjected AAV2/retro-hSyn-Cre-EGFP (AAV2-Retro, titer: 1.02 × 10^13^ vg/ml, OBiO, Shanghai, China) into PrL or ovBNST, and then bilaterally microinjected AAV2/9-EF1α-DIO-hM4Di-mCherry (AAV2/9, titer: 4.83 × 10^12^ vg/ml, OBiO, Shanghai, China) or AAV2/8-EF1α-DIO-mCherry (AAV2/9, titer: 5.04 × 10^12^ vg/ml, OBiO, Shanghai, China) into AI. CNO (5 mg/kg), a synthetic ligand for activating hM4Di in chemogenetic inhibition of neuronal activity, was intraperitoneally administered 30 min prior to the behavioral tests.

For knockdown of DAGLα in AI-innervating PrL or ovBNST neurons, we used our designed DAGLα-sgRNAs AAV vectors [8,12]. Based on CRISPROR (http://crispr.tefor.net/) [52], the DAGLα-sgRNAs were designed to target exon 3 of the *DAGLα* gene. The application of DAGLα-sgRNAs was performed with the following AAV vectors: AAV2/1-hSyn-Cre-mCherry (AAV2/1, titer: 1.67 × 10^13^ vg/ml, OBiO, China) was injected into bilateral AIs, and AAV2/9-hSyn-DIO-SaCas9-3xFLAG (AAV2/9, titer: 1.00 × 10^13^ vg/ml, OBiO, Shanghai, China) and AAV2/9-CMV-EGFP-WPRE-U6-DAGLα-sgRNAs (AAV2/9, titer: 7.23 × 10^12^ vg/ml, OBiO, China) (1:1) were injected into bilateral PrLs or ovBNSTs. AAV2/9-hSyn-DIO-SaCas9-3xFLAG (AAV2/9, titer: 1.00 × 10^13^ vg/ml, OBiO, China) and AAV2/9-CMV-EGFP-WPRE-U6-Scramble (AAV2/9, titer: 7.23 × 10^12^ vg/ml, OBiO, China) (1:1) were injected as the noncoding control. Following a 4-week recovery period, the mice were sacrificed to determine the knockdown efficiency before the establishment of the chronic headache model.

For knockdown of MAGL expression, we employed miR30-short hairpin RNA (shRNA)-mediated RNA interference sequences targeting the *MAGL* mRNA [12]. The *MAGL* mRNA-targeting shRNA sequences were encoded by a Cre-dependent AAV vector: AAV2/9-CaMKIIα-DIO-mCherry-miR30shRNA (MAGL)-WPRE (AAV2/9, titer: 2.00 × 10^12^ vg/ml, OBiO Technology, Shanghai, China). For specific knockdown of MAGL in PrL-projecting or ovBNST-projecting AI neurons, AAV2/retro-hSyn-Cre-EGFP was bilaterally injected into PrL or ovBNST, and then AAV2/9-CaMKIIα-DIO-mCherry-miR30shRNA (MAGL)-WPRE was bilaterally microinjected into AI. AAV2/9-CaMKIIα-DIO-mCherry-miR30shRNA (NC)-WPRE (AAV2/9, titer: 2.00 × 10^12^ vg/ml, OBiO Technology, China) was used as the non-encoding control. Following a 4-week recovery period, the mice were sacrificed to determine the knockdown efficiency before the establishment of the chronic headache model.

For in vivo fiber photometry recording the eCB activity, an AAV vector containing the G protein-coupled receptor activation-based eCB biosensors (GRAB-eCB2.0) (AAV2/9-hSyn-eCB 2.0-GFP) (AAV2/9, titer: 1.00 × 10^12^ vg/ml, Weizhenbio, China) was unilaterally injected into AI (+1.80 mm, +3.00 mm, −2.70 mm). Two weeks later, an optical fiber (outer diameter: 200 μm, numerical aperture: 0.37, Inper, China) was unilaterally implanted above PrL (+1.80 mm, +0.30 mm, −2.10 mm) or ovBNST (+0.30 mm, +0.90 mm, −3.70 mm), ipsilateral to the AI injection site. The optical fiber was fixed to the skull with dental cement.

For in vivo optogenetic activation of CB1R, an AAV vector containing a photosensitive CB1R-based chimeric receptor (AAV2/9-CaMKIIa-opCB1R-mCherry, AAV2/9, titer: 1.00 × 10^12^ vg/ml, OBiO, China) was bilaterally injected into AI. Two weeks later, we bilaterally implanted optical fibers into PrL (+1.80 mm, ±0.30 mm, −2.20 mm) and ovBNST (+0.30 mm, ±0.90 mm, −3.80 mm) to activate the photosensitive CB1Rs expressed in AI terminals within PrL and ovBNST, respectively. Following the implantation, optical fibers were cemented to the skull.

### In vivo optogenetic CB1R activation

Two weeks after the optical fiber implantation, mice underwent 5 d of training to mitigate any maladaptation induced by the testing instruments or environment. For optogenetic activation of CB1R, precise laser stimulation (473 nm, 20 Hz, pulse duration 10 ms, power density at the tip of 4 to 5 mW) was delivered using a laser stimulator.

### In vivo GRAB-eCB2.0 biosensor recording

Following AAV2/9-hSyn-eCB 2.0-GFP virus injection and optical fiber implantations, fluorescent changes from the GRAB-eCB2.0 biosensor were recorded using fiber photometry. The experimental setup employed a commercial fiber photometry system (Nanjing Thinkertech, China) configured according to our established protocol [8,12]. The fluorescence data were imported into MATLAB for further analysis. The temporal values of fluorescence change were presented as Δ*F*/*F*0 (Δ*F* = *F*_t_ − *F*_0_), where *F*_t_ represents the transient fluorescence signal captured during the following 5-s period after Von Frey filament stimuli or open arm entries, and *F*_0_ is the baseline fluorescence intensity measured over the preceding 2 s. The fluorescence signals were then analyzed to assess the time-locked activity of the eCB2.0 biosensor in vAI-PrL or dAI-ovBNST synapses. Exported data underwent statistical analysis and were presented as an average plot and a heatmap.

### Cannula implantation

To inhibit the CB1R of PrL or ovBNST, the stainless steel cannula (outer diameter 0.41 mm, inner diameter 0.25 mm, RWD Life Science, China) was implanted at the top of the bilateral PrL (+1.80 mm, ±0.30 mm, −2.20 mm) or ovBNST (+0.30 mm, ±0.90 mm, −3.80 mm) and chronically fixed to the skull using dental cement. After the pharmacological experiments, a fluorescein isothiocyanate (FITC)-conjugated cholera toxin dye (abs80003, Absin, Shanghai, China) was microinjected to validate the placement.

### Drug administration

For chemogenetic manipulation of neuronal activity, 5 mg/kg [53] of CNO (20230718, Brain Case, China) was intraperitoneally administrated. We performed the behavioral tests 30 min after CNO injection.

For pharmacological inhibition of the CB1R in PrL or ovBNST, 300 nl/side of NESS0327 (0.5 μM, HY-117139, MedChemExpress, USA) was intracerebrally perfused into PrL or ovBNST. The concentration and volume of NESS0327 were determined based on the previous studies [54,55]. The drug delivery was conducted by a perfusion pump (R462, RWD Life Science, China) at a rate of 100 nl/min. Following the drug perfusion, the injection cannula was held for an additional 5 min to prevent the drug backflow.

For pharmacological inhibition of 2-AG hydrolysis, the MAGL inhibitor JZL184 (10 mg/kg, HY-15249, MedChemExpress, USA) was systematically administrated. The dosage of JZL184 was determined according to our previous study [56]. The administration of JZL184 was conducted 10 min after intracerebral injection of NESS0327, and subsequent behavioral tests were performed 2 h after JZL184 injection.

### Tissue processing and immunohistochemistry

Following deep anesthesia, mice were transcardially perfused with ice-cold phosphate-buffered saline (PBS; 0.01 M) followed by 4% paraformaldehyde (PFA). Brains were subsequently postfixed in 4% PFA for 24 h at 4 °C and then cryoprotected in 30% sucrose solution until saturation. Coronal sections (30 μm thickness) were obtained using a cryostat microtome (Leica CM860 UV, Germany). The harvested brain sections were permeabilized with 0.5% Triton X-100 and blocked with 10% donkey serum. For immunofluorescent staining, the sections were incubated in normal donkey serum and primary antibodies for 12 h at 4 °C, followed by the Alexa Fluor 488 or 568 fluorescent secondary antibodies and 4′,6-diamidino-2-phenylindole (DAPI) (P0131, Beyotime Biotechnology, Shanghai, China). Combined RNAscope in situ hybridization with immunostaining was performed to visualize expressions of *Cnr1*, *Slc17a7*, *DAGLα*, and *MAGL* mRNA in labeled neurons. In brief, in situ hybridization was performed in the frozen sections using the RNAscope Fluorescent Multiplex Kit (no. 323100, Advanced Cell Diagnostics, CA, USA) according to the manufacturer’s protocol. The sections were then subjected to the following immunofluorescent staining and mounted with DAPI. The following antibodies for immunofluorescence staining were employed: rabbit anti-c-fos primary antibodies (1:3,000, ab279289, Abcam), rabbit EGFP [1:100, 2555, Cell Signaling Technology (CST)] primary antibodies (1:100, 43590, CST), rabbit anti-mCherry primary antibodies (1:100, 43590, CST), donkey anti-rabbit Alexa Fluor 488 secondary antibodies (1:1,000, ab150113, Abcam), and donkey anti-rabbit Alexa Fluor 568 secondary antibodies (1:1,000, ab175470, Abcam).

Tissue sections were imaged using either a slide-scanning microscope (VS120, Olympus, Tokyo, Japan) or a laser-scanning confocal microscope (FV3000, Olympus, Tokyo, Japan). The locations of regions were determined manually according to the Franklin and Paxinos mouse brain atlas. Data analysis was conducted using National Institutes of Health (NIH) ImageJ software based on 3 sections per animal. Specifically, the threshold was controlled by identical parameters to standardize the detection of the target signals. The quantification of c-fos expression in mCherry-labeled PrL- and ovBNST-projecting AI neurons was calculated as (c-fos^+^mCherry^+^ neurons/mCherry^+^ neurons) × 100. The knockdown efficiency of DAGLα or MGLA was validated by quantifying the *DAGLα* and *MAGL* mRNA relative fluorescent intensity, calculated as the ratio of the total *DAGLα* mRNA^+^EGFP^+^ or *MAGL* mRNA^+^mCherry^+^ pixel value to the total EGFP^+^ or mCherry pixel value within the region of interest (ROI).

### Electrophysiology

Mice transfected with the chemogenetic virus were quickly sacrificed using 4% isoflurane anesthesia, followed by intracardial perfusion of cold oxygenated artificial cerebrospinal fluid (ACSF): 126 mM NaCl, 10 mM glucose, 2.5 mM KCl, 1.25 mM NaH_2_PO4, 25 mM NaHCO_3_, 2 mM MgSO_4_, and 2 mM CaCl_2_. The acute coronal slices (300 μm) containing the AI, PrL, or ovBNST region were obtained using a Leica vibrating microtome (VT1200s, Leica, Germany) and then recovered in oxygenated incubating ACSF (92 mM NaCl, 1.2 mM NaH_2_PO_4_, 2.5 mM KCl, 2 mM CaCl_2_, 20 mM Hepes, 25 mM glucose, 2 mM MgCl_2_, 30 mM NaHCO_3_, 3 mM sodium pyruvate, 5 mM sodium ascorbate) for 30 min at 35 °C and subsequent 30 min at room temperature. After recovery, the slices were placed on the recording chamber to select the target neurons using a microscope (BX51WI, Olympus, Japan) and perfused with the oxygenated ACSF at a rate of 3 ml/min. The pulled patch pipettes (4 to 8 MΩ) were filled with patch pipette solution: 133 mM K-gluconate, 8 mM NaCl, 0.6 mM EGTA, 2 mM Mg·ATP, 0.3 mM Na3·GTP, 10 mM Hepes. Patch-clamp electrophysiology was performed using the Clampfit pClamp 10.0 software and MultiClamp 700B (Molecular Devices, USA).

To validate the inhibitory effects of the chemogenetic virus, a final concentration of 10 μM CNO was perfused in the ACSF. The hM4Di-labeled AI neurons were selected and recorded for at least 2 min during CNO perfusion. The action potential firing rates of hM4Di-labeled AI neurons were then compared before and after CNO perfusion.

To verify the efficacy of the opCB1R virus in vAI→PrL and dAI→ovBNST glutamatergic terminals, postsynaptic recordings in voltage-clamp mode (holding potential of −70 mV) were carried out in the PrL or ovBNST region. The activation of opCB1Rs was triggered with 473-nm blue light from a light-emitting diode (Polygon400, Mightex) delivered through the objective lens, with the light intensity less than 20 mW. We recorded mEPSCs in the presence of tetrodotoxin (1 μM, 554412, Sigma-Aldrich) and picrotoxin (100 μM, HY-101391, MedChemExpress).

### Statistical analysis

Data are presented as mean ± standard error of the mean (SEM). Statistical analyses were performed using SPSS Statistics software (version 17.0; SPSS Inc., USA). Normality of distribution was assessed using the Shapiro–Wilk test, while homogeneity of variances was verified with the Brown–Forsythe test. Normally distributed data with homogeneous variance were analyzed using Student’s or paired *t* tests, and 1- or 2-way analysis of variance (ANOVA) followed by Bonferroni’s post hoc tests. Other datasets were analyzed with a nonparametric test. The level of statistical significance was set at *P* < 0.05.

## Data Availability

All data needed to evaluate the conclusions in the paper are present in the paper and/or the Supplementary Materials.
