# Supplementary material for: Endocannabinoids Block Headache and Anxiety Comorbidity via Two-Pronged Anterior Insular Projections
Source: Research (Wash D C). 2025 Dec 9;8:1031. doi: 10.34133/research.1031 (PMC12688663; doi:10.34133/research.1031)
Supplement: Supplementary 1 — Fig. S1 Table S1 [file research.1031.f1.pdf]

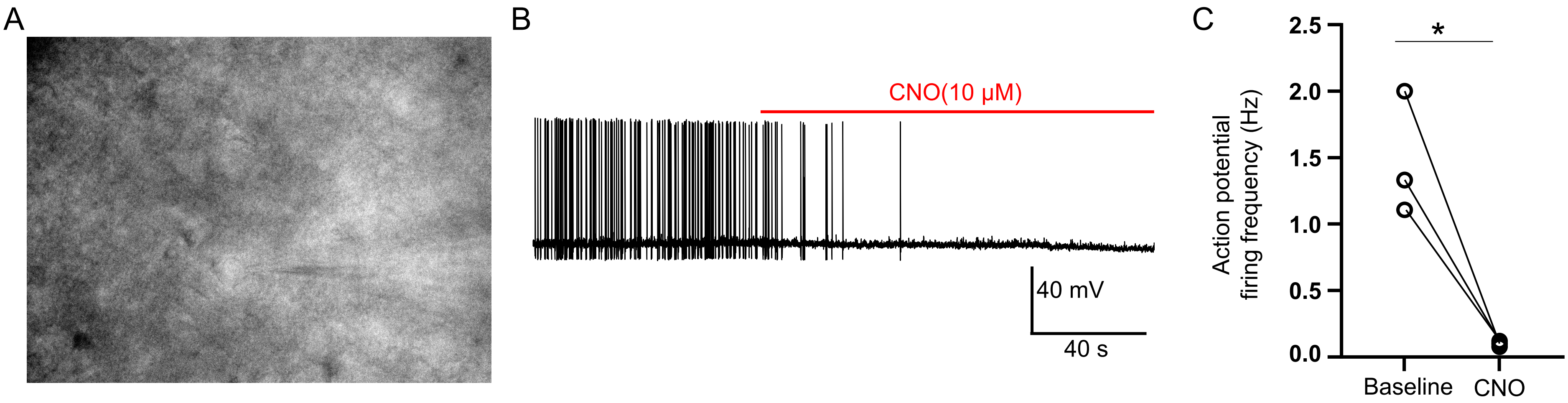

Fig. S1: Validation of the inhibitory effect of chemogenetic strategy on hM4Di-expressing AI neuronal activity. A. Representative image of whole-cell patch-clamp recordings of hM4Di-expressing AI neurons. **B.** Representative firing rate of hM4Di-expressing AI neurons before and after CNO perfusion. **C.** Quantification of the firing rate of hM4Di-expressing AI neurons before and after CNO perfusion. The data are presented as the mean ± SEM, paired t-test, t=5,424, df=2, $P = 0.032$, $^{*}P < 0.05$.

Statistical data

Fig. No	Group	Statistical method	F/t value	*P* value
Fig. 1B	Vehicle vs ISDN	Repeated-measures ANOVA	ISDN: $F_{(1,14)}$=20.356	$P < 0.001$
			Time: $F_{(1,14)}$=17.467	$P < 0.001$
			Interaction: $F_{(1,14)}$=15.414	$P < 0.001$
Fig. 1C	Vehicle vs ISDN	Repeated-measures ANOVA	ISDN: $F_{(1,14)}$=79.742	$P < 0.001$
			Time: $F_{(1,14)}$=6.820	$P < 0.001$
			Interaction: $F_{(1,14)}$=7.640	$P < 0.001$
Fig. 1D	Vehicle vs ISDN	Repeated-measures ANOVA	ISDN: $F_{(1,14)}$=35.025	$P < 0.001$
			Time: $F_{(1,14)}$=7.460	$P < 0.001$
			Interaction: $F_{(1,14)}$=5.941	$P = 0.002$
Fig. 1E	Vehicle vs ISDN	Repeated-measures ANOVA	ISDN: $F_{(1,14)}$=88.728	$P < 0.001$
			Time: $F_{(1,14)}$=8.641	$P < 0.001$
			Interaction: $F_{(1,14)}$=5.667	$P < 0.001$
Fig. 1F	Vehicle vs ISDN	Student's t test	t_{14}=3.211	$P = 0.006$
Fig. 1G	Vehicle vs ISDN	Student's t test	t_{14}=3.617	P = 0.003
Fig. 1H	Vehicle vs ISDN	Student's t test	t_{14}=0.520	P = 0.611
Fig. 1I	Vehicle vs ISDN	Student's t test	t_{14}=2.328	$P = 0.035$
Fig. 1J	Vehicle vs ISDN	Student's t test	t_{14}=2.963	$P = 0.010$
Fig. 1K	Vehicle vs ISDN	Student's t test	t_{14}=0.172	$P = 0.866$
Fig. 1L	Vehicle vs ISDN	Student's t test	t_{14}=3.306	$P = 0.005$
Fig. 1M	Vehicle vs ISDN	Student's t test	t_{14}=4.132	P=0.001
Fig. 1N	Vehicle vs ISDN	Student's t test	t_{14}=1.362	P=0.195
Fig. 1O	Vehicle vs ISDN	Student's t test	t_{14}=2.889	P=0.012
Fig. 1P	Vehicle vs ISDN	Student's t test	t_{14}=3.247	P=0.006
Fig. 1Q	Vehicle vs ISDN	Student's t test	t_{14}=0.783	P=0.447

Fig. No	Group	Statistical method	t value	*P* value
Fig. 2B	Vehicle vs ISDN	Student's t test	t_{10}=11.538	$P < 0.001$
Fig. 2D	Vehicle vs ISDN	Student's t test	t_{10}=6.603	$P < 0.001$
Fig. 2F	Vehicle vs ISDN	Student's t test	t_{10}=7.461	$P < 0.001$
Fig. 2H	Vehicle vs ISDN	Student's t test	t_{10}=13.262	$P < 0.001$
Fig. 2J	Vehicle vs ISDN	Student's t test	t_{10}=14.275	$P < 0.001$

Fig. No	Group	Statistical method	F/t value	*P* value
Fig. 3C	Merged PNs/ mCherry+ PNs vs Merged PNs/ EGFP+ PNs	Student's t test	t_{10}=0.383	$P = 0.710$
Fig. 3F	Vehicle vs ISDN	Two-way ANOVA	ISDN: $F_{(1,14)}$=50.666<br>hM4D: $F_{(1,14)}$=6.634<br>Interaction: $F_{(1,14)}$=12.614	$P < 0.001$<br>$P = 0.022$<br>$P = 0.003$
Fig. 3G	Vehicle vs ISDN	Two-way ANOVA	ISDN: $F_{(1,14)}$=19.735<br>hM4D: $F_{(1,14)}$=0.219<br>Interaction: $F_{(1,14)}$=0.123	$P < 0.001$<br>$P = 0.647$<br>$P = 0.731$
Fig. 3H	Vehicle vs ISDN	Two-way ANOVA	ISDN: $F_{(1,14)}$=18.630<br>hM4D: $F_{(1,14)}$=0.070<br>Interaction: $F_{(1,14)}$=0.090	$P < 0.001$<br>$P = 0.796$<br>$P = 0.768$
Fig. 3I	Vehicle vs ISDN	Two-way ANOVA	ISDN:$F_{(1,14)}$=0.074<br>hM4D: $F_{(1,14)}$=2.606<br>Interaction: $F_{(1,14)}$=0.006	$P = 0.790$<br>$P = 0.129$<br>$P = 0.937$
Fig. 3J	Vehicle vs ISDN	Two-way ANOVA	ISDN: $F_{(1,14)}$=19.60<br>hM4D: $F_{(1,14)}$=0.720<br>Interaction: $F_{(1,14)}$=0.320	$P < 0.001$<br>$P = 0.410$<br>$P = 0.581$
Fig. 3K	Vehicle vs ISDN	Two-way ANOVA	ISDN: $F_{(1,14)}$=26.107<br>hM4D: $F_{(1,14)}$=0.800<br>Interaction: $F_{(1,14)}$=0.017	P < 0.001<br>$P = 0.386$<br>$P = 0.899$
Fig. 3L	Vehicle vs ISDN	Two-way ANOVA	ISDN: $F_{(1,14)}$=2.013	$P = 0.178$

			hM4D: $F_{(1,14)}$=2.375	*P* = 0.146
			Interaction: $F_{(1,14)}$=0.641	*P* = 0.437
Fig. 3O	Vehicle vs ISDN	Two-way ANOVA	ISDN: $F_{(1,14)}$=83.870	*P* < 0.001
			hM4D: $F_{(1,14)}$=0.09	*P* = 0.761
			Interaction: $F_{(1,14)}$=0.772	*P* = 0.395
Fig. 3P	Vehicle vs ISDN	repeated-measures ANOVA	ISDN: $F_{(1,14)}$=9.124	*P* = 0.009
		Two-way ANOVA	hM4D: $F_{(1,14)}$=16.251	*P* = 0.001
			Interaction: $F_{(1,14)}$=2.881	*P* = 0.112
Fig. 3Q	Vehicle vs ISDN	Two-way ANOVA	ISDN: $F_{(1,14)}$=15.916	*P* = 0.001
			hM4D: $F_{(1,14)}$=1.909	*P* = 0.189
			Interaction: $F_{(1,14)}$=4.820	*P* = 0.045
Fig. 3R	Vehicle vs ISDN	Two-way ANOVA	ISDN: $F_{(1,14)}$=0.616	*P* = 0.446
			hM4D: $F_{(1,14)}$=2.289	*P* = 0.153
			Interaction: $F_{(1,14)}$=0.042	*P* = 0.841
Fig. 3S	Vehicle vs ISDN	Two-way ANOVA	ISDN: $F_{(1,14)}$=11.055	*P* = 0.005
			hM4D: $F_{(1,14)}$=2.121	*P* = 0.167
			Interaction: $F_{(1,14)}$=3.055	*P* = 0.102
Fig. 3T	Vehicle vs ISDN	Two-way ANOVA	ISDN: $F_{(1,14)}$=12.157	*P* = 0.004
			hM4D: $F_{(1,14)}$=3.184	*P* = 0.096
			Interaction: $F_{(1,14)}$=4.059	*P* = 0.064
Fig. 3U	Vehicle vs ISDN	Two-way ANOVA	ISDN: $F_{(1,14)}$=3.908	*P* = 0.068
			hM4D: $F_{(1,14)}$=0.743	*P* = 0.403
			Interaction: $F_{(1,14)}$=0.775	*P* = 0.394

Fig. No	Group	Statistical method	t value	*P* value
Fig. 4L	Vehicle vs ISDN	Student's t test	t_{10}=7.777	*P* < 0.001
Fig. 4P	Vehicle vs ISDN	Student's t test	t_{10}=0.851	*P* = 0.414
Fig. 4U	Vehicle vs ISDN	Student's t test	t_{10}=5.563	*P* < 0.001
Fig. 4Y	Vehicle vs ISDN	Student's t test	t_{10}=6.737	*P* < 0.001

Fig. No	Group	Statistical method	F/t value	*P* value
Fig. 5F	DAGLα NC vs DAGLα KD	Student's t test	t_{10}=15.117	*P* < 0.001
Fig. 5G	Vehicle vs ISDN	Two-way ANOVA	ISDN: $F_{(1,14)}$=162.842	*P* < 0.001
			DAGLα: $F_{(1,14)}$=2.934	*P* = 0.109
			Interaction: $F_{(1,14)}$=0.132	*P* = 0.722
Fig. 5H	Vehicle vs ISDN	Two-way ANOVA	ISDN: $F_{(1,14)}$=17.640	*P* < 0.001
			DAGLα: $F_{(1,14)}$=5.879	*P* = 0.029
			Interaction: $F_{(1,14)}$=0.097	*P* = 0.760
Fig. 5I	Vehicle vs ISDN	Two-way ANOVA	ISDN: $F_{(1,14)}$=28.921	*P* < 0.001
			DAGLα: $F_{(1,14)}$=2.334	*P* = 0.149
			Interaction: $F_{(1,14)}$=0.233	*P* = 0.637
Fig. 5J	Vehicle vs ISDN	Two-way ANOVA	ISDN: $F_{(1,14)}$=0.133	*P* = 0.721
			DAGLα: $F_{(1,14)}$=1.881	*P* = 0.192
			Interaction: $F_{(1,14)}$=0.350	*P* = 0.564
Fig. 5K	Vehicle vs ISDN	Two-way ANOVA	ISDN: $F_{(1,14)}$=18.050	*P* < 0.001
			DAGLα: $F_{(1,14)}$=2.446	*P* = 0.140
			Interaction: $F_{(1,14)}$=0.212	*P* = 0.653
Fig. 5L	Vehicle vs ISDN	Two-way ANOVA	ISDN: $F_{(1,14)}$=18.713	*P* < 0.001
			DAGLα: $F_{(1,14)}$=2.394	*P* = 0.144
			Interaction: $F_{(1,14)}$=0.020	*P* = 0.888
Fig. 5M	Vehicle vs ISDN	Two-way ANOVA	ISDN: $F_{(1,14)}$=1.936	*P* = 0.186
			DAGLα: $F_{(1,14)}$=0.341	*P* = 0.569
			Interaction: $F_{(1,14)}$=723	*P* = 0.409
Fig. 5S	DAGLα NC vs DAGLα KD	Student's t test	t_{10}=11.637	*P* < 0.001
Fig. 5T	Vehicle vs ISDN	Two-way ANOVA	ISDN: $F_{(1,13)}$=199.871	*P* < 0.001
			DAGLα: $F_{(1,13)}$=2.988	*P* = 0.108
			Interaction: $F_{(1,13)}$=0.022	*P* = 0.885
Fig. 5U	Vehicle vs ISDN	Two-way ANOVA	ISDN: $F_{(1,13)}$=31.814	*P* < 0.001
			DAGLα: $F_{(1,13)}$=1.647	*P* = 0.222
			Interaction: $F_{(1,13)}$=0.001	*P* = 0.981
Fig. 5V	Vehicle vs ISDN	Two-way ANOVA	ISDN: $F_{(1,13)}$=25.790	*P* < 0.001
			DAGLα: $F_{(1,13)}$=4.353	*P* = 0.057

			Interaction: $F_{(1,13)}$=0.042	*P* = 0.840
Fig.5W	Vehicle vs ISDN	Two-way ANOVA	ISDN: $F_{(1,13)}$=1.353	*P* = 0.266
			DAGLα: $F_{(1,13)}$=1.262	*P* = 0.282
			Interaction: $F_{(1,13)}$=0.368	*P* = 0.554
Fig. 5X	Vehicle vs ISDN	Two-way ANOVA	ISDN: $F_{(1,13)}$=40.054	*P* < 0.001
			DAGLα: $F_{(1,13)}$=0.004	*P* = 0.952
			Interaction: $F_{(1,13)}$=0.857	*P* = 0.372
Fig. 5Y	Vehicle vs ISDN	Two-way ANOVA	ISDN: $F_{(1,13)}$=44.153	*P* < 0.001
			DAGLα: $F_{(1,13)}$=0.503	*P* = 0.491
			Interaction: $F_{(1,13)}$=0.857	*P* = 0.088
Fig. 5Z	Vehicle vs ISDN	Two-way ANOVA	ISDN: $F_{(1,13)}$=0.039	*P* = 0.847
			DAGLα: $F_{(1,13)}$=0.343	*P* = 0.568
			Interaction: $F_{(1,13)}$=0.103	*P* = 0.753

Fig. No	Group	Statistical method	t value	*P* value
Fig. 6F	MAGL NC vs MAGL KD	Student's t test	t_{10}=14.866	*P* < 0.001
Fig. 6G	Vehicle vs ISDN	Two-way ANOVA	ISDN: $F_{(1,14)}$=123.265	*P* < 0.001
			MAGL: $F_{(1,14)}$=2.856	*P* = 0.113
			interaction: $F_{(1,14)}$=10.763	*P* = 0.005
Fig. 6H	Vehicle vs ISDN	Two-way ANOVA	ISDN: $F_{(1,14)}$=21.832	*P* < 0.001
			MAGL: $F_{(1,14)}$=0.014	*P* = 0.907
			interaction: $F_{(1,14)}$=0.126	*P* = 0.728
Fig. 6I	Vehicle vs ISDN	Two-way ANOVA	ISDN: $F_{(1,14)}$=26.358	*P* < 0.001
			MAGL: $F_{(1,14)}$=0.053	*P* = 0.821
			interaction: $F_{(1,14)}$=0.053	*P* = 0.818
Fig. 6J	Vehicle vs ISDN	Two-way ANOVA	ISDN: $F_{(1,14)}$=0.235	*P* = 0.635
			MAGL: $F_{(1,14)}$=0.029	*P* = 0.868
			interaction: $F_{(1,14)}$=0.193	*P* = 0.067
Fig. 6K	Vehicle vs ISDN	Two-way ANOVA	ISDN: $F_{(1,14)}$=16.580	*P* < 0.001
			MAGL: $F_{(1,14)}$=0.307	*P* = 0.589
			interaction: $F_{(1,14)}$=0.006	*P* = 0.938
Fig. 6L	Vehicle vs ISDN	Two-way ANOVA	ISDN: $F_{(1,14)}$=25.275	*P* < 0.001

			MAGL: $F_{(1,14)}$=0.182	*P* = 0.676
			interaction: $F_{(1,14)}$=4.530	*P* = 0.052
Fig. 6M	Vehicle vs ISDN	Two-way ANOVA	ISDN: $F_{(1,14)}$=0.164	*P* = 0.692
			MAGL: $F_{(1,14)}$=1.705	*P* = 0.213
			interaction: $F_{(1,14)}$=0.001	*P* = 0.985
Fig. 6S	MAGL NC vs MAGL KD	Student's t test	t_{10}=14.104	*P* < 0.001
Fig. 6T	Vehicle vs ISDN	Two-way ANOVA	ISDN: $F_{(1,14)}$=203.448	*P* < 0.001
			MAGL: $F_{(1,14)}$=0.266	*P* = 0.614
			interaction: $F_{(1,14)}$=0.013	*P* = 0.912
Fig. 6U	Vehicle vs ISDN	Two-way ANOVA	ISDN: $F_{(1,14)}$=6.986	*P* = 0.019
			MAGL: $F_{(1,14)}$=2.340	*P* = 0.148
			interaction: $F_{(1,14)}$=5.347	*P* = 0.036
Fig. 6V	Vehicle vs ISDN	Two-way ANOVA	ISDN: $F_{(1,14)}$=7.004	*P* = 0.019
			MAGL: $F_{(1,14)}$=9.488	*P* = 0.008
			interaction: $F_{(1,14)}$=5.656	*P* = 0.032
Fig. 6W	Vehicle vs ISDN	Two-way ANOVA	ISDN: $F_{(1,14)}$=1.718	*P* = 0.211
			MAGL: $F_{(1,14)}$=0.170	*P* = 0.686
			interaction: $F_{(1,14)}$=0.374	*P* = 0.550
Fig. 6X	Vehicle vs ISDN	Two-way ANOVA	ISDN: $F_{(1,14)}$=15.867	*P* = 0.001
			MAGL: $F_{(1,14)}$=1.298	*P* = 0.274
			interaction: $F_{(1,14)}$=10.867	*P* = 0.005
Fig. 6Y	Vehicle vs ISDN	Two-way ANOVA	ISDN: $F_{(1,14)}$=12.200	*P* = 0.004
			MAGL: $F_{(1,14)}$=4.205	*P* = 0.060
			interaction: $F_{(1,14)}$=35.389	P < 0.001
Fig. 6Z	Vehicle vs ISDN	Two-way ANOVA	ISDN: $F_{(1,14)}$=1.429	*P* = 0.252
			MAGL: $F_{(1,14)}$=0.049	*P* = 0.828
			interaction: $F_{(1,14)}$=0.354	*P* = 0.561

Fig. No	Group	Statistical method		t value	*P* value
Fig. 7F	Control vs Light	Paired t test		t_6=4.354	*P* = 0.005
Fig. 7G	Control vs Light	Paired t test		t_6=0.5113	*P* = 0.627
Fig. 7I	mCherry vs opCB1R	Student's t test	off	t_{14}=0.180	*P* = 0.860
			on	t_{14}=3.704	*P* = 0.002
			off	t_{14}=0.549	*P* = 0.592
Fig. 7J	mCherry vs opCB1R	Student's t test	off	t_{14}=0.517	*P* = 0.613
			on	t_{14}=1.974	*P* = 0.068
			off	t_{14}=0.393	*P* = 0.700
Fig. 7K	mCherry vs opCB1R	Student's t test	off	t_{14}=0.388	*P* = 0.704
			on	t_{14}=1.175	*P* = 0.260
			off	t_{14}=0.343	*P* = 0.737
Fig. 7L	mCherry vs opCB1R	Student's t test	off	t_{14}=0.788	*P* = 0.444
			on	t_{14}=1.276	*P* = 0.223
			off	t_{14}=0.332	*P* = 0.745
Fig. 7M	mCherry vs opCB1R	Student's t test	off	t_{14}=0.991	*P* = 0.339
			on	t_{14}=0.264	*P* = 0.796
			off	t_{14}=0.560	*P* = 0.584
Fig. 7S	Control vs Light	Paired t test		t_5=4.357	*P* = 0.007
Fig. 7T	Control vs Light	Paired t test		t_5=1.386	*P* = 0.225
Fig. 7V	mCherry vs opCB1R	Student's t test	off	t_{14}=0.713	*P* = 0.488
			on	t_{14}=0.709	*P* = 0.490
			off	t_{14}=0.291	*P* = 0.775
Fig. 7W	mCherry vs opCB1R	Student's t test	off	t_{14}=0.123	*P* = 0.903
			on	t_{14}=2.833	*P* = 0.013
			off	t_{14}=0.271	*P* = 0.790
Fig. 7X	mCherry vs opCB1R	Student's t test	off	t_{14}=1.088	*P* = 0.295
			on	t_{14}=3.350	*P* = 0.005
			off	t_{14}=0.319	*P* = 0.754
Fig. 7Y	mCherry vs opCB1R	Student's t test	off	t_{14}=1.723	*P* = 0.107
			on	t_{14}=2.468	*P* = 0.027

			off	t_{14}=1.265	*P* = 0.227
Fig. 7Z	mCherry vs opCB1R	Student's t test	off	t_{14}=1.843	*P* = 0.087
			on	t_{14}=3.635	*P* = 0.003
			off	t_{14}=0.411	*P* = 0.687

Fig. No	Statistical method	F value	*P* value
Fig. 8D	One-way ANOVA	$F_{(3,30)}$ =54.513	$P < 0.001$
Fig. 8E	One-way ANOVA	$F_{(3,30)}$ =5.668	*P*=0.003
Fig. 8F	One-way ANOVA	$F_{(3,30)}$ =5.259	*P*=0.005
Fig. 8G	One-way ANOVA	$F_{(3,30)}$ =1.994	*P*=0.136
Fig. 8H	One-way ANOVA	$F_{(3,30)}$ =4.343	*P*=0.012
Fig. 8I	One-way ANOVA	$F_{(3,30)}$ =10.722	$P < 0.001$
Fig. 8J	One-way ANOVA	$F_{(3,30)}$ =0.534	*P*=0.663
Fig. 8N	One-way ANOVA	$F_{(3,30)}$ =26.673	$P < 0.001$
Fig. 8O	One-way ANOVA	$F_{(3,30)}$ =7.955	$P < 0.001$
Fig. 8P	One-way ANOVA	$F_{(3,30)}$ =11.665	$P < 0.001$
Fig. 8Q	One-way ANOVA	$F_{(3,30)}$ =0.940	*P*=0.434
Fig. 8R	One-way ANOVA	$F_{(3,30)}$ =7.922	$P < 0.001$
Fig. 8S	One-way ANOVA	$F_{(3,30)}$ =8.303	$P < 0.001$
Fig. 8T	One-way ANOVA	$F_{(3,30)}$ =1.589	*P*=0.213
